# 5-Aminolevulinic Acid Ameliorates Chronic Experimental Autoimmune Neuritis Through a Dual Mechanism of Mitochondrial Protection and Immunomodulation

**DOI:** 10.3390/ijms26178512

**Published:** 2025-09-02

**Authors:** Shingo Konno, Takafumi Uchi, Hideo Kihara, Toshiki Fujioka

**Affiliations:** Department of Neurology, Toho University Ohashi Medical Center, Tokyo 153-8515, Japan; takafumi.uchi@med.toho-u.ac.jp (T.U.); hideo.kihara@med.toho-u.ac.jp (H.K.);

**Keywords:** chronic inflammatory demyelinating polyneuropathy (CIDP), 5-aminolevulinic acid (5-ALA), experimental autoimmune neuritis (EAN), mitochondria, neuroprotection

## Abstract

Chronic inflammatory demyelinating polyneuropathy (CIDP) is an autoimmune disorder characterized by inflammation and neurodegeneration, yet current therapies lack direct neuroprotective effects. We investigated the therapeutic potential of 5-aminolevulinic acid (5-ALA), a key precursor for mitochondrial heme synthesis, in a chronic experimental autoimmune neuritis (EAN) rat model of CIDP. Rats with established EAN received daily oral 5-ALA (100 mg/kg) or vehicle. Treatment efficacy was assessed by clinical scoring, nerve histopathology, and biochemical analyses of sciatic nerves. 5-ALA administration significantly ameliorated clinical disease severity. This was associated with local immunomodulation in the sciatic nerve, marked by reduced pro-inflammatory IFN-γ and increased anti-inflammatory IL-10 levels. Concurrently, 5-ALA exerted direct neuroprotective effects, evidenced by restored mitochondrial ATP production, decreased oxidative DNA damage, upregulated antioxidant heme oxygenase-1 (HO-1), and improved myelin sheath integrity. These findings suggest that 5-ALA may offer a dual therapeutic benefit by targeting both local inflammation and mitochondrial-mediated neuroprotection. By addressing key pathological mechanisms currently unmet by standard therapies, 5-ALA emerges as a promising disease-modifying candidate for CIDP.

## 1. Introduction

Chronic inflammatory demyelinating polyneuropathy (CIDP) is an autoimmune disorder of the peripheral nervous system, whose pathological hallmarks include endoneurial infiltration of inflammatory cells led by macrophages, the involvement of CD4+ and CD8+ T-cells, and the formation of “onion bulbs” indicative of recurrent demyelination and remyelination [1]. Investigating this complex pathology and evaluating new therapies depends on animal models that faithfully replicate the human disease [1,2,3]. Unlike acute experimental autoimmune neuritis (EAN), which models Guillain-Barré syndrome, recently developed chronic EAN models effectively mirror the clinical and histological features of human CIDP, including a sustained or relapsing clinical course, demyelination with axonal injury, and a late-phase accumulation of T-cells and macrophages involving IL-17 [2,4]. Consequently, chronic EAN is widely recognized as a reliable preclinical platform for assessing novel therapeutic strategies for CIDP [2,4].

While current first-line treatments for CIDP—such as immunoglobulins, corticosteroids, and plasma exchange—focus on immunosuppression, they face considerable limitations, including treatment resistance in some patients, significant side effects, and high economic costs [5,6]. A more fundamental issue is that these therapies target the inflammatory process but do not directly address the irreversible axonal damage that is a primary determinant of long-term disability [5,6]. In various neuropathies, mitochondrial dysfunction is a key pathogenic mechanism [7]. Given the high energy requirements of peripheral nerves, mitochondrial impairment can lead to a bioenergetic crisis, increased oxidative stress, and apoptosis-mediated neurodegeneration [7]. Therefore, therapeutic strategies that not only modulate the immune response but also preserve axonal integrity by maintaining mitochondrial health represent a critical and currently unmet need in CIDP management [5,6,7].

To address this therapeutic gap, this study evaluates 5-aminolevulinic acid (5-ALA), a known enhancer of mitochondrial metabolism, as a novel therapeutic candidate. The objective was to investigate the efficacy of 5-ALA in a chronic EAN rat model that reproduces the key pathological features of human CIDP. We hypothesized that 5-ALA would exert multifaceted neuroprotective effects, including local immunomodulation, preservation of mitochondrial function, and stabilization of myelin architecture through activation of the antioxidant response. To test this hypothesis, we administered daily oral 5-ALA to rats with chronic EAN and comprehensively evaluated its effects on clinical severity, nerve histopathology, and various immunological and metabolic markers.

## 2. Results

### 2.1. 5-ALA Ameliorates Clinical Severity of EAN

**Figure 1:**
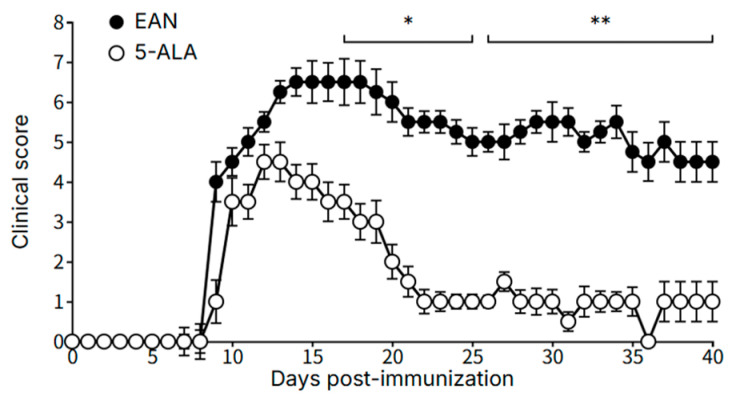
5-ALA administration ameliorates the clinical severity of EAN. Daily clinical scores of EAN rats treated with vehicle or 5-ALA (100 mg/kg/day) from day 7 to day 39 post-immunization. Data are presented as mean ± SEM (n = 5 per group). Statistical significance was determined using the Mann–Whitney U test. * *p* < 0.05, ** *p* < 0.01 vs. EAN group.

Rats immunized with the palmitoylated P0 peptide universally developed clinical signs of EAN, with an onset around day 11 p.i. for both groups. Control rats progressed to a severe disease state, reaching a peak mean clinical score of 7.0 ± 0.3 on day 15. In contrast, daily oral administration of 5-ALA, initiated on day 7, significantly ameliorated the clinical severity of EAN. A significant difference between the groups emerged on day 17 (*p* < 0.05) and was maintained throughout the observation period, with the level of significance increasing in later stages of the disease (*p* < 0.01) (Figure 1).

### 2.2. 5-ALA Modulates the Local Inflammatory Milieu in Sciatic Nerves

**Figure 2:**
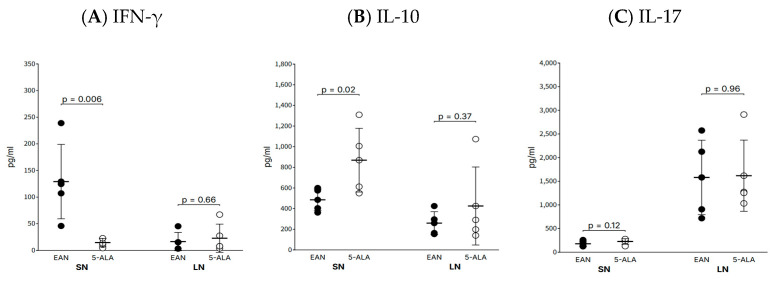
5-ALA modulates the local inflammatory cytokine profile in the sciatic nerve. Concentrations of IFN-γ (**A**), IL-10 (**B**), and IL-17A (**C**) in the s sciatic nerve (SN) and draining lymph nodes (LN; inguinal). at day 40 post-immunization, measured by ELISA. Data are presented as mean ± SEM (n = 5 per group). Statistical significance was determined using the Mann–Whitney U test. Abbreviations: SN = sciatic nerve; LN = lymph node.

To investigate the effect of 5-ALA on the immune response, cytokine levels in the sciatic nerves and draining lymph nodes were measured at day 40 p.i. (Figure 2). In the sciatic nerves, 5-ALA treatment led to a significant reduction in the levels of the pro-inflammatory cytokine IFN-γ (14.3 ± 7.9 pg/mL) compared to the control group (128.8 ± 69.8 pg/mL; *p* = 0.006). Conversely, the levels of the anti-inflammatory cytokine IL-10 were significantly increased in the 5-ALA-treated group (867.8 ± 137.9 pg/mL) versus the control group (483.9 ± 46.4 pg/mL; *p* = 0.02). No significant difference was observed in the levels of IL-17A between the two groups (*p* = 0.12). In the draining lymph nodes, concentrations of IFN-γ, IL-10, and IL-17A did not differ significantly between the groups (*p* > 0.05 for all).

### 2.3. 5-ALA Preserves Mitochondrial Function and Enhances Antioxidant Response

**Figure 3:**
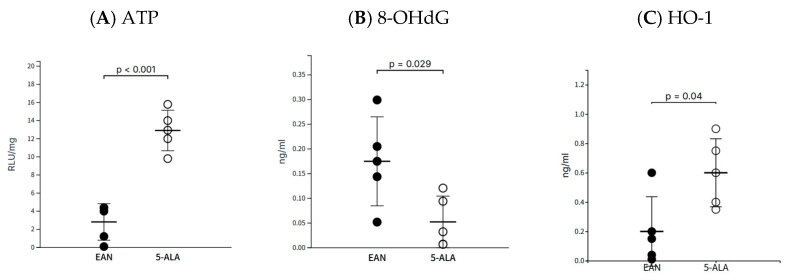
5-ALA preserves mitochondrial function and enhances antioxidant response in the sciatic nerve. Biochemical analysis of sciatic nerves at day 40 post-immunization. (**A**) ATP levels expressed as Relative Light Units (RLU) normalized to total protein. (**B**) 8-OHdG levels as a marker of oxidative DNA damage. (**C**) Protein levels of the antioxidant enzyme HO-1. Data are presented as mean ± SEM (n = 5 per group). Statistical significance was determined using the Mann–Whitney U test.

We next assessed markers of mitochondrial function and oxidative stress in the sciatic nerves (Figure 3). 5-ALA administration resulted in markedly elevated ATP levels compared to the control group (12.9 ± 2.23 vs. 2.81 ± 2.01 RLU/mL; *p* < 0.001). Furthermore, 5-ALA treatment significantly reduced levels of 8-OHdG, a marker of oxidative DNA damage (0.05 ± 0.02 vs. 0.17 ± 0.10 ng/mL; *p* = 0.029). Consistent with an enhanced antioxidant response, protein levels of HO-1 were also significantly higher in the 5-ALA group compared to controls (0.60 ± 0.12 vs. 0.20 ± 0.11 ng/mL; *p* = 0.04).

### 2.4. 5-ALA Improves Myelin Integrity in Chronic EAN

**Figure 4:**
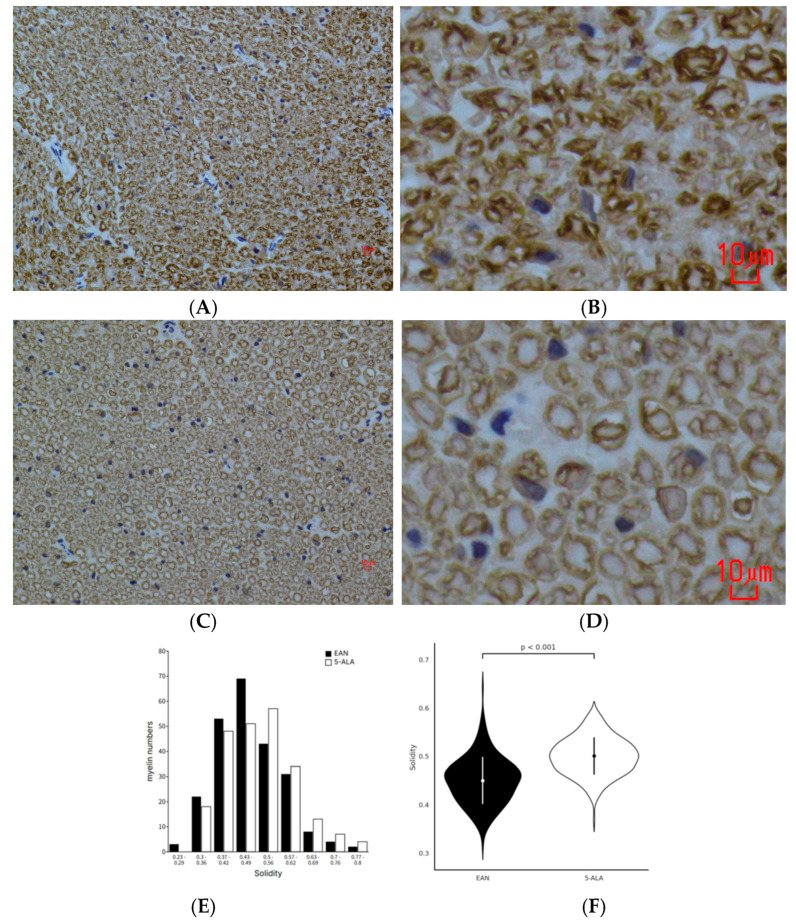
5-ALA treatment improves myelin integrity in the sciatic nerves of EAN rats. (**A**) Representative image of a Myelin Protein Zero (MPZ)-stained sciatic nerve cross-section at day 40 from a control EAN rat. The cellular infiltration and signs of demyelination. Scale bar = 10 µm. (**B**) Representative image of an MPZ-stained sciatic nerve cross-section at day 40 from a 5-ALA-treated rat, showing reduced cellular infiltration and more preserved myelin structure. Scale bar = 10 µm. To further clarify the morphological changes in myelin architecture, we included high-magnification MPZ-stained images from the same samples. These provide a more detailed view of myelin sheath compactness and fragmentation. In the EAN group, fibers often showed irregular contours and disrupted wrapping (**C**), while the 5-ALA-treated group exhibited more uniform, concentric lamellae, consistent with the solidity index improvement (**D**). Quantification of myelin integrity via the “solidity” index, defined as the ratio of a fiber’s area to its convex hull area (Solidity = Area/Convex Area). Solidity reflects the compactness of individual myelinated fibers, with a value of 1.0 indicating structurally intact myelin and lower values reflecting demyelination. (**E**) The histogram shows the distribution of solidity values from 235 myelinated fibers per group, binned into 9 intervals to visualize differences in structural integrity. (**F**) Violin plot of the same solidity values from 235 fibers per group, illustrating distributional shifts between groups. The EAN group (black) shows a broader and left-shifted distribution compared to the 5-ALA group (white), indicating lower myelin integrity. Dots and error bars denote group means ± standard deviations. Mann–Whitney U test.

Histological examination of sciatic nerves at day 40 revealed demyelination and inflammatory cell infiltration in the control EAN group (Figure 4A,B). In contrast, these pathological changes were visibly ameliorated in 5-ALA-treated animals (Figure 4C,D). To quantify myelin integrity, the “solidity” of MPZ-stained myelinated fibers was calculated, where a higher value indicates a more regular, intact myelin sheath. The analysis showed that the mean solidity index was significantly higher in the 5-ALA group than in the control group (*p* = 0.04 showed in violin plot), confirming improved myelin architecture following treatment (Figure 4E,F).

## 3. Discussion

This study provides the first evidence that daily oral administration of 5-ALA exerts significant therapeutic effects in a chronic EAN model of CIDP. The treatment not only ameliorated the clinical severity of the disease but also modulated the local inflammatory environment within the peripheral nerves, characterized by a significant reduction in pro-inflammatory IFN-γ and an increase in anti-inflammatory IL-10. Concurrent with this immunomodulatory effect, 5-ALA was associated with a neuroprotective effect, potentially linked to preserved mitochondrial function, an enhanced antioxidant response via HO-1 upregulation, and improved structural integrity of the myelin sheath. These findings are significant as they suggest that 5-ALA targets both the inflammatory and the neurodegenerative aspects of the disease, addressing a critical gap in current CIDP therapies that are largely limited to immunosuppression.

One of the key findings of this study is the significant modulation of the local inflammatory environment within the sciatic nerve by 5-ALA. Specifically, it suppressed the expression of the pro-inflammatory T helper 1 (Th1)-derived cytokine IFN-γ while increasing the expression of the traditionally anti-inflammatory cytokine IL-10. The reduction in IFN-γ aligns with the observed clinical improvement and is consistent with reports that 5-ALA can directly suppress the macrophage inflammatory response via HO-1 induction [8]. As macrophages are key drivers of T-cell responses, this pathway likely contributed to the decrease in IFN-γ production. Nevertheless, we acknowledge that the role of IL-10 in CIDP is complex, and the observed increase may not necessarily reflect a purely beneficial effect. Functional validation through targeted pathway inhibition will be required in future studies. While IL-10 typically increases as a regulatory response following an IFN-γ peak in EAN [9], recent studies have suggested a paradoxical, pathogenic role for IL-10 in CIDP by promoting T-cell migration into peripheral nerves via S1PR1 [10]. That the disease improved in our study despite elevated IL-10 suggests that the net benefit of 5-ALA’s potent local anti-inflammatory and neuroprotective actions may outweigh the potential cell-trafficking effect of IL-10. It is also possible that 5-ALA, via HO-1, promotes a regulatory M2 macrophage phenotype, and the resulting IL-10 functions in a different context than that which promotes pathogenic T-cell migration.

While our study measured IFN-γ, IL-10, and IL-17A, we acknowledge that more extensive cytokine profiling and immunohistochemical quantification of infiltrating immune cells would provide additional insight into the immunomodulatory effects of 5-ALA. These remain important directions for future studies.

The therapeutic effects of 5-ALA observed in this study were not limited to immunomodulation. The marked recovery of ATP levels and the reduction in the oxidative DNA damage marker 8-OHdG in sciatic nerve tissue indicate that 5-ALA directly protects mitochondrial bioenergetics and mitigates oxidative stress. This functional improvement was further supported by the upregulation of the antioxidant enzyme HO-1, and ultimately manifested as improved structural integrity of the myelin sheath. While our current data do not include direct measurements of heme, PpIX, or heme-related enzymatic activities such as cytochrome c oxidase or δ-aminolevulinate synthase, the observed improvements in ATP levels and reduction of 8-OHdG strongly suggest enhancement of mitochondrial function. Given the known biochemical role of 5-ALA as a rate-limiting precursor in the heme biosynthetic pathway, it is plausible that its administration improved electron transport chain activity via increased heme availability. These changes are consistent with a possible augmentation of heme-dependent processes, but remain indirect and speculative. Importantly, all animals in our study were maintained under tightly controlled ambient light conditions, effectively eliminating the possibility of photodynamic activation of any PpIX formed. Under such low-light conditions, PpIX—if produced—would remain photodynamically inert and function only as a biosynthetic intermediate. This controlled environment ensures that the observed effects of 5-ALA are attributable to metabolic modulation rather than light-induced cytotoxicity. Therefore, the beneficial effects observed in our study, particularly the restoration of ATP levels and the reduction in oxidative DNA damage, are more likely attributable to enhanced mitochondrial metabolic support rather than light-induced cytotoxicity. Nonetheless, we acknowledge that definitive validation of this proposed mechanism will require direct quantification of PpIX, heme levels, and related enzymatic activities in peripheral nerve tissue. These measurements—e.g., via liquid chromatography–mass spectrometry or enzyme activity assays—are planned as part of future mechanistic investigations. We have now explicitly noted the absence of these measurements as a limitation of the present study.

This strategy of targeting mitochondrial health is an established therapeutic concept in peripheral neuropathies. For example, α-lipoic acid exerts neuroprotection by rescuing mitochondrial membrane potential and inducing protective proteins like frataxin [11], while acetyl-L-carnitine supports energy metabolism by enhancing fatty acid oxidation and ATP production [11,12]. These “mitochondrial nutrients” have confirmed efficacy in conditions like diabetic and chemotherapy-induced neuropathy, often with minimal side effects [12,13]. However, while these agents act primarily as antioxidants or metabolic cofactors, 5-ALA, as a precursor for heme synthesis, may restore mitochondrial homeostasis at a more fundamental level by directly providing an essential component for the electron transport chain. This direct neuroprotective action, combined with its immunomodulatory effects, makes 5-ALA a particularly compelling therapeutic candidate for a multifactorial disease like CIDP.

The findings of this study carry significant implications for the future therapeutic landscape of CIDP, suggesting a potential shift from purely immunosuppressive strategies to a dual-pronged approach that also incorporates direct neuroprotection. Current first-line therapies are often unable to prevent the cumulative disability that results from ongoing axonal degeneration, highlighting a critical unmet need for treatments that can protect and repair nervous tissue, not just dampen the immune attack [5]. Our demonstration that 5-ALA simultaneously modulates the local inflammatory environment and enhances mitochondrial health offers a proof-of-concept for such a strategy. By targeting the bioenergetic and oxidative stress pathways within nerve cells, 5-ALA represents a move towards a disease-modifying therapy aimed at neuronal preservation. Furthermore, as an orally administered agent with a favorable safety profile, it holds the potential to significantly improve patient quality of life and reduce the substantial economic burden associated with current infusion-based treatments [5]. Ultimately, integrating metabolic and neuroprotective interventions into clinical practice could shift the therapeutic paradigm for CIDP from simple disease control towards neuronal repair and functional restoration.

In summary, this study demonstrates that 5-ALA is a promising therapeutic agent for autoimmune demyelinating neuropathy, acting through a novel dual mechanism of local immunomodulation and direct mitochondrial-mediated neuroprotection. In response to the observed trends in mitochondrial rescue and inflammatory modulation, we proposed a dual mechanism model for 5-ALA’s action. To visually support this interpretation, we added a schematic (Graphical abstract) summarizing the immune-metabolic framework underlying the observed effects. Although our mechanistic dataset is limited to three primary markers (ATP, 8-OHdG, HO-1), these were selected to represent distinct arms of mitochondrial and antioxidant regulation. The consistency among these endpoints provides preliminary but meaningful support for our hypothesis.

However, several limitations should be considered. All experiments were conducted exclusively in female Lewis rats, which may limit the generalizability of our findings across sexes. Additionally, therapeutic effects were evaluated at a single time point (day 40 post-immunization), precluding insights into the temporal dynamics of disease progression and recovery. While we observed increased HO-1 and IL-10 expression, our study design does not permit mechanistic validation of their causal roles in the observed neuroprotection. Furthermore, as this study is based on a preclinical EAN model, its translatability to the clinical heterogeneity of human CIDP remains uncertain.

Future research should therefore focus on longitudinal analyses, mechanistic studies, and ultimately, clinical trials to confirm the safety and efficacy of 5-ALA in CIDP patients. Another important aspect for future investigation is the pharmacokinetics and tissue distribution of 5-ALA. Although 5-ALA was administered via oral gavage in this study to standardize systemic exposure, interindividual differences in absorption and metabolism remain a concern for clinical translation. Moreover, while our study did not include direct measurement of 5-ALA or PpIX in sciatic nerves, the use of low-light housing conditions ensured that any tissue accumulation occurred within a metabolic—not photodynamic—context. To substantiate the therapeutic rationale, future studies should incorporate liquid chromatography–mass spectrometry or radiolabel-based methods to profile 5-ALA and its metabolites in peripheral nerve tissue, especially under disease conditions. We have revised the manuscript to clearly state this limitation and include pharmacokinetic evaluation as a recommended direction for further preclinical development.

The identification of biomarkers to monitor neuroprotective responses—an unmet need in the field [5]—would be crucial for stratifying patients and optimizing therapeutic outcomes. In addition, evaluating 5-ALA as an adjunct to existing immunotherapies may offer a synergistic strategy that not only controls inflammation but also promotes neuronal resilience and repair. By shifting the therapeutic paradigm to encompass metabolic enhancement and cellular bioenergetics, this work lays a foundation for a new class of reparative treatments in CIDP and other neuroinflammatory disorders.

## 4. Materials and Methods

### 4.1. Ethical Statement

All animal experiments were approved by the Toho University’s Animal Experiment Committee on 1 April 2025 (protocol number: 24-567) and were conducted in accordance with the regulations and guidelines of the committee.

### 4.2. Animals

Female Lewis rats aged 7 weeks were purchased from Charles River Japan (Yokohama, Japan). They were housed in the Toho University Ohashi Experimental Animal Laboratory, and every effort was made to minimize their suffering. Animals were housed in individually ventilated cages under standardized environmental conditions at the Toho University Ohashi Animal Laboratory, including a 12 h light–dark cycle. Ambient lighting was measured to remain consistently on 135 lux throughout the experiment. Given this verified low-light intensity, phototoxic accumulation of Protoporphyrin IX (PpIX) following 5-ALA administration is unlikely to Room temperature and humidity were controlled at 22 ± 2 °C and 50 ± 10%, respectively. Animals had ad libitum access to CRF-1 chow and autoclaved water.

### 4.3. Induction of Chronic Experimental Autoimmune Neuritis (Chronic EAN)

EAN was induced using methods previously described to generate a chronic EAN model with palmitoylated peptide [9,14]. Briefly, rats were subcutaneously immunized at the base of the tail with a 200 µL injection containing 200 µg of a synthetic palmitoylated P0 peptide (amino acids 180–199, sequence: H-AC(Palm)KRGRQTPVLYAMLDHSRS-OH; BEX Co., Ltd., Tokyo, Japan) emulsified in an equal volume of Complete Freund’s Adjuvant (CFA; Sigma-Aldrich, St. Louis, MO, USA). The immunization was performed under light anesthesia with sevoflurane (Mylan, Osaka, Japan).

### 4.4. Experimental Groups and 5-ALA Administration

The rats were randomly divided into two groups (n = 5 per group): a control group (Vehicle) and a 5-ALA group. From day 7 to day 39 post-immunization (p.i.), rats in the 5-ALA group received a daily administration of 5-ALA (100 mg/kg) dissolved in purified water via oral gavage. The control group received an equivalent volume of purified water following the same schedule.

### 4.5. Clinical Assessment

The clinical severity of EAN was assessed daily and scored on a 0–9 point scale, calculated as the sum of scores from the tail, forelimbs, and one hindlimb. Motor function was scored as follows: for the tail, 0 = no clinical sign, 1 = paralysis of the tail tip, 2 = incomplete paralysis of the entire tail, and 3 = complete paralysis of the entire tail; for the forelimbs, 0 = no clinical sign, 1 = unable to climb a fence, 2 = unable to walk, and 3 = complete paralysis; and for the hindlimb, 0 = no clinical sign, 1 = toe paralysis only, 2 = incomplete foot dorsiflexion while walking, and 3 = complete paralysis (drags leg).

### 4.6. Tissue Collection and Preparation

On day 40 p.i., rats were deeply anesthetized with sevoflurane and transcardially perfused with ice-cold phosphate-buffered saline (PBS). The sciatic nerves and draining inguinal lymph nodes were collected. For ATP measurement, a portion of the tissues was immediately snap-frozen in liquid nitrogen and stored at −80 °C. Samples for 8-hydroxy-2′-deoxyguanosine (8-OHdG), Heme Oxygenase-1 (HO-1), and cytokine analyses were preserved in Allprotect Tissue Reagent (Qiagen, Hilden, Germany) and stored in a refrigerator. For histological analysis, the remaining tissue portions were fixed in 10% neutral buffered formalin.

### 4.7. Quantification of Cytokines

Tissue homogenates were prepared from approximately 1.5 cm segments of sciatic nerve and inguinal lymph nodes (approximately 3 mm in diameter) in 1.5 mL of phosphate-buffered saline (PBS) using a bead-based homogenizer. Supernatants were assayed for IFN-γ, IL-10, and IL-17A using commercial sandwich ELISA kits (R&D Systems, Minneapolis, MN, USA; #DY585, #DY522, #DY8410, respectively) according to the manufacturer’s instructions. Absorbance was measured using a Bio-Rad microplate reader. Cytokine concentrations were normalized to total protein content, determined with a Qubit 2.0 Fluorometer, and expressed as pg/mL.

### 4.8. Assessment of Mitochondrial Function and Oxidative Stress

Oxidative DNA damage was assessed by measuring 8-OHdG in tissue supernatants with a competitive ELISA kit (Japan Institute for the Control of Aging, Shizuoka, Japan). HO-1 levels were quantified using a rat-specific ELISA kit (Enzo Life Sciences, Inc., Farmingdale, NY, USA). To measure ATP, frozen sciatic nerve tissue was homogenized in PBS, and luminescence was immediately quantified using an AquaSnap Total ATP test kit and a SystemSURE Plus luminometer. ATP levels were reported as Relative Light Units (RLU) normalized to total protein content.

### 4.9. Histological Analysis and Myelin Quantification

Formalin-fixed, paraffin-embedded sciatic nerve sections were immunostained for Myelin Protein Zero (MPZ) (1:100; Sigma-Aldrich, #ABN363) following heat-induced antigen retrieval. The signal was detected using a polymer-based HRP system and visualized with diaminobenzidine (DAB).

To quantify myelin integrity, we measured the solidity of individual MPZ-positive myelinated fibers, defined as the ratio of the fiber’s area to the area of its convex hull (Solidity = Area/Convex Area). This geometric shape descriptor reflects the structural compactness and regularity of myelin profiles, with a value of 1.0 indicating a smooth, convex sheath, and lower values suggesting irregular, concave, or fragmented structures typical of demyelination. Digital images of sciatic nerve cross-sections were captured at 40× magnification and subjected to background subtraction and Otsu’s thresholding for binary segmentation. Myelin profiles were then extracted using custom Python scripts (Python v3.10) incorporating the OpenCV (v4.8) and scikit-image (v0.21) libraries. For each group (EAN and 5-ALA), 235 fibers were randomly selected from MPZ-stained sections (n = 5 rats per group). Only fibers ≥ 20 pixels in area and with solidity values between 0.2 and 1.0 were included in the analysis to ensure morphological relevance. Solidity distributions were visualized using both histograms (binned into 9 intervals) and violin plots. Group means and standard deviations were overlaid as red dot-error markers.

### 4.10. Statistical Analysis

All statistical analyses were performed with EZR (Saitama Medical Center, Jichi Medical University, Saitama, Japan), which is a graphical user interface for R (The R Foundation for Statistical Computing, Vienna, Austria) [15]. Data are presented as mean ± SEM. All between-group comparisons were evaluated using the two-tailed Mann–Whitney U test. A *p*-value < 0.05 was considered statistically significant. This study was designed as a hypothesis-generating pilot investigation with limited statistical power. Although the Mann–Whitney U test was used to accommodate small sample sizes (n = 5 per group), future studies with larger cohorts are needed to confirm these findings.

## Data Availability

The datasets used in this study are available from the corresponding author on reasonable request.
